# Reducing Delayed Discharges After Neck of Femur Fracture Surgery in a Major Trauma Centre: A Retrospective Quality Improvement Study

**DOI:** 10.7759/cureus.110386

**Published:** 2026-06-07

**Authors:** Hamza Ahmed, Aima Gilani, Aarish Azeem, Marium Rizwan, Muhammad Hassaan, Abed Alfattah Mahmoud Alnsour, Mohamed Said Ammar

**Affiliations:** 1 Trauma and Orthopaedics, Salford Royal NHS Foundation Trust, Manchester, GBR; 2 Emergency Assessment Unit, Salford Royal NHS Foundation Trust, Manchester, GBR; 3 Trauma and Orthopaedics and Spinal Surgery, Salford Royal NHS Foundation Trust, Manchester, GBR

**Keywords:** delayed discharge, discharge planning, fracture neck of femur, hip fracture, length of stay, medically fit for discharge, orthogeriatrics

## Abstract

Background

Delays between medical readiness for discharge and actual hospital discharge are a common challenge after neck of femur fracture surgery. These delays can prolong hospital stay, increase exposure to hospital-associated complications, occupy acute beds unnecessarily, and create additional distress for older patients and their families. Although hip fracture pathways often focus on time to surgery and orthogeriatric review, the discharge phase is less frequently examined as a target for local quality improvement.

Methods

We performed a retrospective before-and-after quality improvement study at a United Kingdom major trauma centre. Consecutive adult patients who underwent surgery for a neck of femur fracture between January 2025 and December 2025 were included. In July 2025, a multidisciplinary discharge-readiness bundle was introduced. This included setting an estimated date of discharge within 24 hours, daily criteria-led board rounds, early triggers for occupational therapy and social work input, escalation of weekend physiotherapy, pharmacy prompts for discharge prescriptions, and twice-weekly escalation meetings with community partners. The primary outcome was delayed discharge, defined as discharge occurring more than 48 hours after the recorded medically fit for discharge (MFFD) date. Secondary outcomes included the MFFD-to-discharge interval, postoperative length of stay, total length of stay, discharge destination, 30-day readmission, and inpatient mortality.

Results

Overall, 134 surgical neck of femur fracture cases were assessed, with 67 patients in each study period. Baseline patient characteristics were similar between groups. Following implementation of the bundle, delayed discharge decreased from 43/67 cases (64.2%) to 24/67 cases (35.8%) (odds ratio: 3.21, 95% confidence interval: 1.58-6.50; p = 0.002). The mean MFFD-to-discharge interval fell from 5.2 to 2.7 days (p < 0.001). Postoperative length of stay decreased from 18.9 to 13.7 days (p = 0.003), while total length of stay decreased from 21.4 to 16.1 days (p = 0.007). Discharge to the patient’s usual residence increased from 44.8% to 62.7%. There was no observed increase in 30-day readmission or inpatient mortality.

Conclusions

Introduction of a multidisciplinary discharge-readiness bundle was associated with a meaningful reduction in delayed discharge after neck of femur fracture surgery at a major trauma centre. The intervention was low-cost, practical to implement, and was not associated with an increase in readmission or inpatient mortality. Further prospective evaluation should include balancing measures, patient-reported outcomes, and measures of community care capacity.

## Introduction

Hip fracture is one of the most significant injuries seen in older adult trauma [1]. It is common, time-critical, and often marks a major turning point in a patient’s health. Many patients experience a loss of mobility, independence, or confidence after the injury, and a substantial number require new or increased support after discharge. Neck of femur fractures, therefore, account for a large part of the operative fragility-fracture workload in major trauma centres. Much of the improvement work in hip fracture care has understandably focused on the early part of the pathway: rapid diagnosis, adequate analgesia, admission to an orthopaedic ward, timely surgery, orthogeriatric review, and early mobilisation. These steps remain central to good care. However, for many patients and families, the most difficult part of the pathway begins once the operation is complete and the patient is approaching discharge.

NICE guidance on hip fracture management emphasises the importance of a coordinated multidisciplinary hip fracture programme, extending from admission through to the patient’s return to the community [2]. National audit has also moved beyond a narrow focus on mortality and time to theatre, placing greater emphasis on mobilisation, rehabilitation, and return home. The National Hip Fracture Database (NHFD) 2025 annual report noted that more than 70,000 people sustain a hip fracture each year in England, Wales, and Northern Ireland [3]. It also highlighted that recovery should be assessed by whether patients reach the right place of care, are helped to get up after surgery, and receive the support they need to return home. The report further linked weekend physiotherapy provision with shorter hospital stays and recommended appropriate physiotherapy capacity throughout the week.

In practice, discharge after hip fracture surgery is rarely straightforward, especially in a major trauma centre [4]. Patients are often frail, have multiple comorbidities, or live with cognitive impairment. Many also depend on several services inside and outside the hospital. Even after the fracture has been treated and acute medical issues have settled, discharge may still be held up by therapy assessments, equipment, care packages, community hospital or rehabilitation-bed availability, transport, discharge prescriptions, family discussions, or mental capacity and best-interest processes [5]. These delays can create a gap between the date a patient is recorded as medically fit for discharge (MFFD) and the date they actually leave the acute hospital.

That gap is not just an administrative inconvenience. For older patients, additional days in hospital can mean further deconditioning, delirium, falls, pressure injury, poor sleep, infection, and loss of confidence. For the hospital, delayed discharge reduces acute bed capacity, increases the likelihood of patients being cared for outside the most appropriate ward area, and can affect the flow of other urgent orthopaedic trauma patients [6,7]. Recent cohort studies have shown that delayed discharge after hip fracture surgery is common and is shaped by a mixture of patient factors, clinical complexity, pre-fracture residence, and organisational barriers [8]. Importantly, some of these barriers can be addressed if discharge planning starts early and is treated as a shared clinical responsibility rather than something to be dealt with once a patient is already medically fit.

At our centre, delayed discharge after neck of femur fracture surgery was repeatedly raised during daily trauma bed meetings as both a patient-safety and operational issue. Informal review of local practice suggested that discharge planning often gathered pace only after a patient had been declared medically fit. By that stage, outstanding therapy, social-care, and pharmacy tasks could already have created avoidable delay. We therefore introduced a pragmatic multidisciplinary discharge-readiness bundle. The aim was to make discharge planning visible from admission, support earlier escalation, and reduce the delay after MFFD without increasing unsafe discharge, readmission, or mortality.

This study evaluated whether the discharge-readiness bundle reduced delayed discharge after neck of femur fracture surgery at a major trauma centre. The primary objective was to compare the proportion of patients discharged more than 48 hours after MFFD before and after implementation. Secondary objectives included MFFD-to-discharge delay, postoperative and total length of stay, discharge destination, 30-day readmission, and inpatient mortality.

## Materials and methods

Study design and setting

This was a single-centre, retrospective before-and-after quality improvement study carried out at a United Kingdom major trauma centre. The centre has an established hip fracture pathway, with daily orthopaedic trauma operating capacity, orthogeriatric input, physiotherapy, occupational therapy, pharmacy support, discharge coordination, and working links with community rehabilitation and social-care teams.

Participants

All consecutive adult patients who underwent surgery for a neck of femur fracture between 1 January 2025 and 31 December 2025 were screened for inclusion. Patients discharged between January and June 2025 formed the pre-intervention cohort, while those discharged between July and December 2025 formed the post-intervention cohort.

Patients were eligible if they were aged 18 years or older, had a radiologically confirmed intracapsular or extracapsular neck of femur fracture, underwent operative treatment during the index admission, and were discharged from the major trauma centre within the study period. Patients were excluded if they were managed non-operatively, had a periprosthetic fracture, had a pathological fracture related to malignancy, were transferred out before surgery, died before an MFFD date could be assigned, or had incomplete documentation of either MFFD or discharge date.

Intervention

The multidisciplinary discharge-readiness bundle was introduced on 1 July 2025. It was developed after reviewing delayed discharge cases, mapping common points of delay, and gathering feedback from the wider team, including orthopaedics, orthogeriatrics, nursing, physiotherapy, occupational therapy, pharmacy, discharge coordinators, and site management.

The bundle included six core elements. First, an estimated date of discharge was recorded within 24 hours of admission and reviewed again after surgery. Second, the daily ward round included clear documentation of the criteria that needed to be met before discharge. Third, occupational therapy screening and equipment needs were identified on postoperative day 1, where clinically appropriate. Fourth, patients who were unlikely to return home without additional support were referred to social work or the discharge coordination team by postoperative day 2. Fifth, pharmacy teams were prompted to prepare discharge prescriptions at least 24 hours before the expected discharge date. Finally, patients with anticipated complex discharge needs were discussed at twice-weekly escalation huddles with community rehabilitation and social-care representatives. Weekend physiotherapy escalation was also introduced for patients whose discharge was likely to depend on further mobility progression or stair assessment.

Outcomes

The primary outcome was delayed discharge, defined as discharge occurring more than 48 hours after the recorded MFFD date. MFFD was taken as the first date on which the orthopaedic and orthogeriatric teams documented that the patient no longer required acute inpatient care. At this point, discharge was considered appropriate once any remaining care arrangements, equipment, rehabilitation placement, transport, or family discussions had been completed.

Secondary outcomes included the number of days between MFFD and actual discharge, postoperative length of stay, total length of stay, discharge destination, discharge to the patient’s usual residence, readmission to the trust within 30 days, and inpatient mortality. The balancing measures were 30-day readmission and inpatient mortality.

Data sources and variables

Data were collected from several routinely used clinical systems, including the electronic patient record, theatre management system, discharge summaries, therapy notes, and the discharge-coordinator database. Extracted variables included age, sex, pre-fracture residence, fracture type, operation type, American Society of Anesthesiologists (ASA) grade, Clinical Frailty Scale (CFS), Abbreviated Mental Test Score (AMTS) or documented cognitive impairment, time to surgery, mobilisation within 24 hours after surgery, weekend physiotherapy input, MFFD date, discharge date, discharge destination, readmission, and mortality.

To support data quality, a random 10% sample of records was independently reviewed by a second reviewer for accuracy.

Statistical analysis

Categorical variables were reported as counts and percentages. Continuous variables were summarised using the mean and standard deviation when they were approximately normally distributed, and the median and interquartile range when the distribution was skewed.

Comparisons between the pre- and post-intervention cohorts were made using Fisher’s exact test or the chi-square test for categorical variables, the independent samples t-test for normally distributed continuous variables, and the Mann-Whitney U test for skewed variables. The relationship between the intervention period and delayed discharge was reported as an odds ratio with a 95% confidence interval. Statistical significance was defined as p < 0.05.

All analyses were performed using standard statistical software. Descriptive statistics were rounded to one decimal place, and p-values were rounded to three decimal places.

## Results

A total of 151 patients with a neck of femur fracture were screened during the 2025 study period. Seventeen patients were excluded: six were managed non-operatively, four had periprosthetic fractures, three were transferred to another provider before definitive surgery, two died before an MFFD date could be assigned, and two had incomplete discharge-date documentation.

The final study population included 134 operative cases, divided equally between the pre-intervention and post-intervention cohorts, with 67 patients in each group.

Baseline characteristics were broadly similar between the two cohorts (Table 1). The mean age was 82.1 years in the pre-intervention cohort and 81.5 years in the post-intervention cohort. Around one-third of patients in each group had documented dementia or cognitive impairment, and the median Clinical Frailty Scale score was 5 in both periods. Surgery within 36 hours was achieved in 76.1% of patients before the intervention and 80.6% after the intervention.

**Table 1:** Baseline patient and operative characteristics.

Characteristic	Pre-intervention (n = 67)	Post-intervention (n = 67)	Test statistic/p-value
Age, mean (SD), years	82.1 (8.9)	81.5 (9.4)	t = 0.38; p = 0.704
Female sex, n (%)	48 (71.6)	46 (68.7)	χ² = 0.04; p = 0.849
Admission from own home/sheltered housing, n (%)	52 (77.6)	54 (80.6)	χ² = 0.05; p = 0.829
Care-home resident pre-fracture, n (%)	15 (22.4)	13 (19.4)	χ² = 0.05; p = 0.829
Documented dementia/cognitive impairment, n (%)	21 (31.3)	22 (32.8)	χ² = 0.00; p = 1.000
Clinical Frailty Scale, median (IQR)	5 (4-6)	5 (4-6)	p = 0.882
ASA grade III-IV, n (%)	46 (68.7)	44 (65.7)	χ² = 0.03; p = 0.852
Intracapsular fracture, n (%)	34 (50.7)	32 (47.8)	χ² = 0.03; p = 0.864
Extracapsular fracture, n (%)	33 (49.3)	35 (52.2)	χ² = 0.03; p = 0.864
Hemiarthroplasty, n (%)	29 (43.3)	27 (40.3)	χ² = 0.03; p = 0.864
Dynamic hip screw or intramedullary nail, n (%)	38 (56.7)	40 (59.7)	χ² = 0.03; p = 0.864
Surgery within 36 hours, n (%)	51 (76.1)	54 (80.6)	χ² = 0.18; p = 0.673

The primary outcome improved following implementation of the discharge-readiness bundle (Table 2). The proportion of patients discharged more than 48 hours after being recorded as MFFD fell from 43/67 (64.2%) in the pre-intervention cohort to 24/67 (35.8%) in the post-intervention cohort. Patients in the pre-intervention period had significantly higher odds of delayed discharge than those treated after implementation of the bundle (OR: 3.21, 95% CI: 1.58-6.50; χ² = 9.67; p = 0.002). The mean delay between MFFD and actual discharge decreased by 2.5 days, from 5.2 to 2.7 days (t = 3.63; p < 0.001).

**Table 2:** Discharge process measures and clinical outcomes. MFFD: medically fit for discharge

Outcome	Pre-intervention (n = 67)	Post-intervention (n = 67)	Effect estimate/test statistic/p-value
Delayed discharge >48h after MFFD, n (%)	43 (64.2)	24 (35.8)	OR 3.21, 95% CI 1.58-6.50; χ² = 9.67; p = 0.002
MFFD-to-discharge delay, mean (SD), days	5.2 (4.7)	2.7 (3.1)	t = 3.63; p < 0.001
MFFD-to-discharge delay, median (IQR), days	4.0 (2.0-8.0)	2.0 (1.0-4.0)	p < 0.001
Postoperative length of stay, mean (SD), days	18.9 (11.3)	13.7 (8.5)	t = 3.01; p = 0.003
Total length of stay, mean (SD), days	21.4 (12.6)	16.1 (9.4)	t = 2.76; p = 0.007
Mobilised by postoperative day 1, n (%)	41 (61.2)	50 (74.6)	χ² = 2.19; p = 0.135
Weekend physiotherapy input when inpatient over weekend, n/N (%)	11/57 (19.3)	34/56 (60.7)	χ² = 18.53; p < 0.001
Discharged to usual residence, n (%)	30 (44.8)	42 (62.7)	χ² = 3.63; p = 0.056
Discharged to community hospital/rehabilitation bed, n (%)	24 (35.8)	17 (25.4)	χ² = 1.27; p = 0.255
New discharge to care home, n (%)	8 (11.9)	4 (6.0)	χ² = 0.82; p = 0.365
30-day readmission, n (%)	5 (7.5)	6 (9.0)	χ² = 0.00; p = 1.000
Inpatient mortality, n (%)	3 (4.5)	2 (3.0)	χ² = 0.00; p = 1.000

Length of stay also improved after implementation (Table 2). Mean postoperative length of stay decreased from 18.9 to 13.7 days, representing a reduction of 5.2 days (t = 3.01; p = 0.003). Mean total length of stay decreased from 21.4 to 16.1 days, a reduction of 5.3 days (t = 2.76; p = 0.007). Among patients who remained in hospital over a weekend, exposure to weekend physiotherapy increased from 19.3% to 60.7% (χ² = 18.53; p < 0.001).

Discharge to the patient’s usual residence increased from 44.8% to 62.7% (Table 2). This approached, but did not meet, conventional statistical significance (χ² = 3.63; p = 0.056). There was no observed increase in 30-day readmission (χ² = 0.00; p = 1.000) or inpatient mortality (χ² = 0.00; p = 1.000).

The most commonly documented reasons for delayed discharge were waits for packages of care, limited community rehabilitation capacity, and outstanding therapy-related actions (Table 3). The greatest proportional reduction was seen in therapy- and equipment-related delays, which were consistent with the earlier occupational therapy screening and escalation of weekend physiotherapy introduced as part of the bundle. Despite the intervention, social-care capacity remained the most frequent ongoing reason for delayed discharge.

**Table 3:** Documented primary reason for delayed discharge among patients delayed >48 hours after MFFD. MFFD: medically fit for discharge

Reason for delay	Pre-intervention (n = 43)	Post-intervention (n = 24)
Awaiting package of care/social-care capacity	14 (32.6%)	7 (29.2%)
Awaiting community hospital or rehabilitation bed	10 (23.3%)	5 (20.8%)
Awaiting therapy assessment, stairs assessment, or equipment	8 (18.6%)	3 (12.5%)
Awaiting family meeting, mental-capacity process, or best-interest decision	5 (11.6%)	4 (16.7%)
Awaiting transport or out-of-area coordination	3 (7.0%)	3 (12.5%)
Awaiting discharge prescription or medication reconciliation	3 (7.0%)	2 (8.3%)

**Figure 1:**
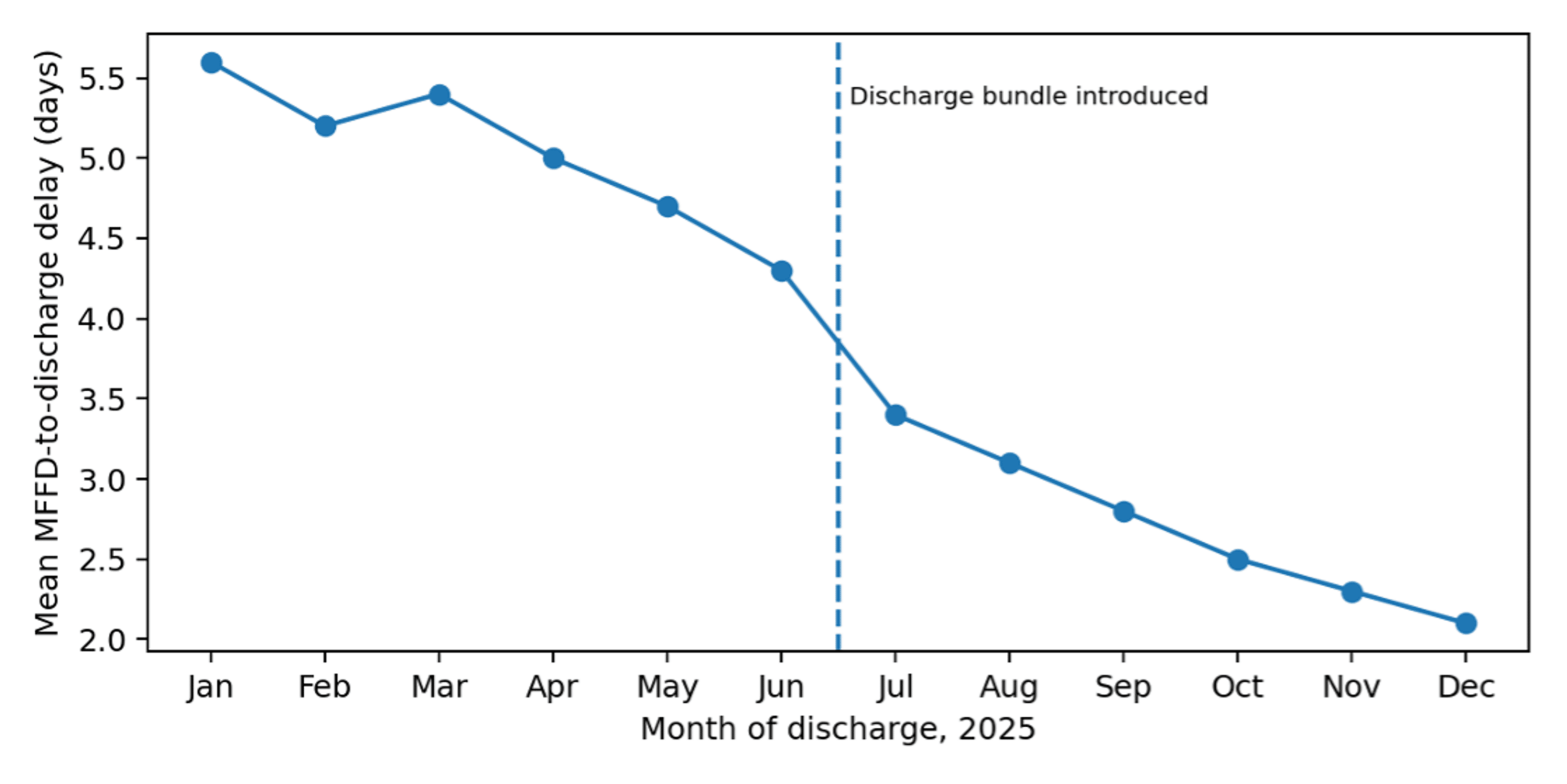
Monthly mean delay from MFFD to actual discharge. MFFD: medically fit for discharge

The run chart (Figure 1) shows a gradual reduction in MFFD-to-discharge delay after the discharge-readiness bundle was introduced in July 2025.

**Figure 2:**
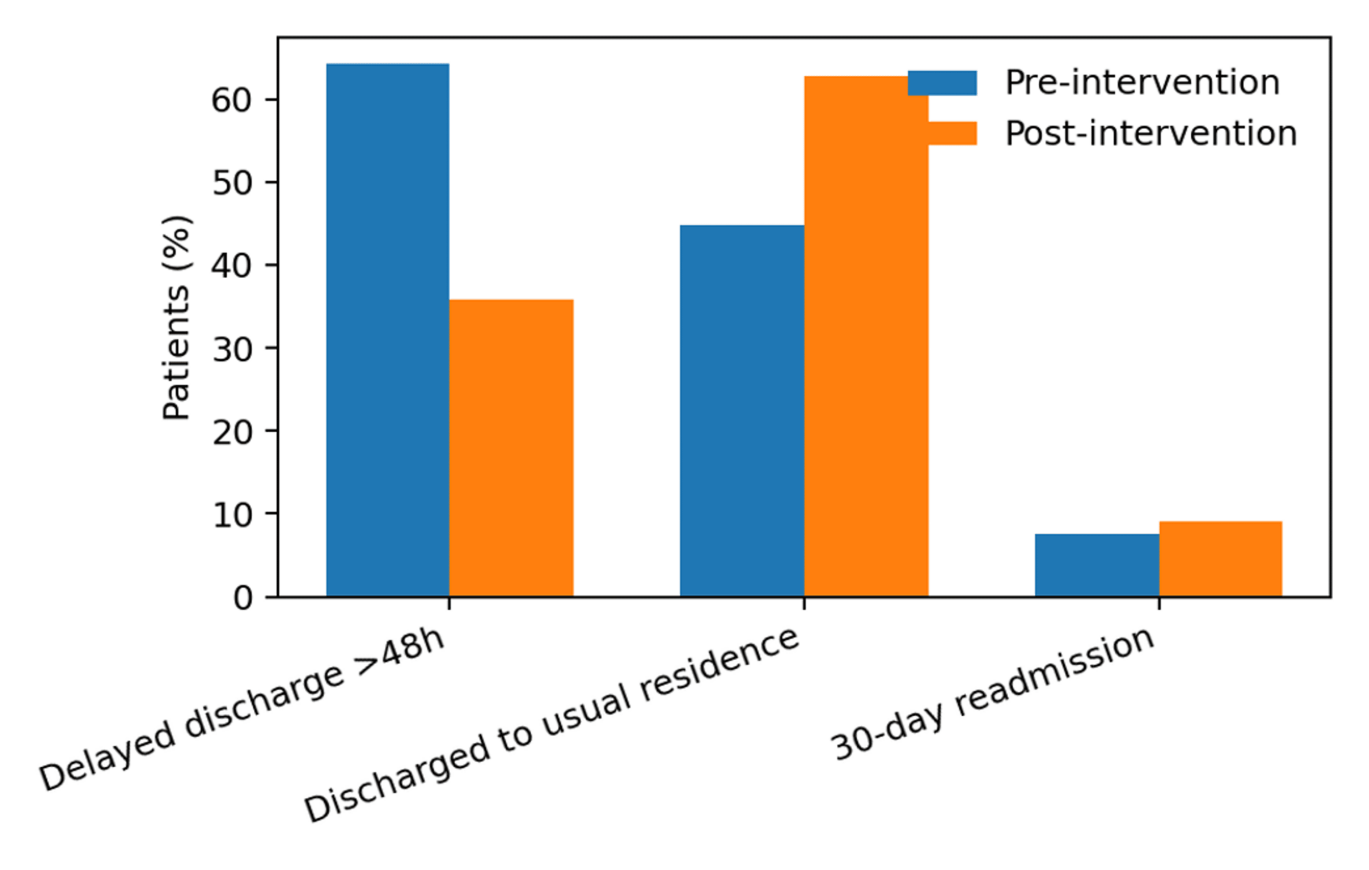
Key outcomes before and after the discharge-readiness bundle.

Implementation was associated with fewer delayed discharges and a higher proportion of patients returning to their usual residence, while 30-day readmission remained broadly similar (Figure 2).

## Discussion

In this retrospective study of 134 operative neck of femur fracture cases at a major trauma centre, implementation of a multidisciplinary discharge-readiness bundle was associated with a marked reduction in delayed discharge. The proportion of patients discharged more than 48 hours after being recorded as MFFD fell from 64.2% to 35.8%, and the average delay between MFFD and discharge decreased by 2.5 days. These changes were accompanied by shorter postoperative and total hospital stay, greater weekend physiotherapy exposure, and a higher proportion of patients returning to their usual residence. Reassuringly, there was no observed increase in 30-day readmission or inpatient mortality, suggesting that shorter delays were not achieved at the expense of patient safety.

These findings reinforce the idea that discharge after hip fracture surgery should be viewed as part of the clinical pathway, rather than as a separate administrative step at the end of admission [9]. Many of the factors that delay discharge are visible early in the patient journey. These include baseline dependency, cognitive impairment, pre-fracture residence, stairs at home, equipment needs, family or carer availability, social-care capacity, and the likely need for further rehabilitation [10]. The bundle did not introduce a new clinical treatment. Instead, it aimed to bring existing tasks forward, clarify what needed to happen before discharge, and create a more consistent process for escalating complex cases. This approach is in keeping with the wider national emphasis on coordinated multidisciplinary hip fracture care and timely rehabilitation [11].

Our findings are also consistent with previous work showing that orthogeriatric and multidisciplinary models can reduce length of stay and improve process outcomes after hip fracture [12,13]. However, the persistence of social-care and rehabilitation-bed delays after implementation is important. It highlights that acute hospital teams can improve internal processes, but they cannot fully control the wider discharge pathway. For this reason, major trauma centre improvement work should involve community partners wherever possible and should measure the specific reasons for delay, rather than relying on length of stay alone.

The most visible improvement in our pathway appeared to relate to earlier therapy and equipment planning [14]. Therapy-related delays were reduced after the introduction of postoperative day 1 occupational therapy screening, clearer stair-assessment planning, and weekend physiotherapy escalation. This is clinically plausible. Many hip fracture patients need a relatively small number of discharge-enabling steps, such as safe transfer practice, mobility progression, stair assessment, toileting and washing assessment, equipment delivery, and family or carer education. If these tasks are only addressed once the patient is medically fit, discharge is almost inevitably delayed. When they run alongside medical recovery, discharge can take place closer to the point at which acute inpatient care is no longer required.

Although the increase in discharge to the usual residence did not reach conventional statistical significance, the direction of change is clinically important. Returning to the usual place of residence is a meaningful outcome for patients and families, and may capture recovery more directly than length of stay alone [15]. Even so, higher rates of discharge home should be interpreted carefully. They should be considered alongside readmission, falls, carer strain, patient confidence, and access to therapy or support after discharge. Future evaluations should therefore include patient and carer experience, functional recovery, and post-discharge support measures.

This study has several practical implications. First, hip fracture ward rounds should include an anticipated discharge date and clear discharge criteria from an early stage. Second, therapy and social-care referrals should be triggered by discharge risk rather than waiting until the patient is formally declared medically fit. Third, weekend rehabilitation should be targeted to patients for whom therapy input is likely to support discharge or prevent avoidable functional decline. Fourth, delayed discharge dashboards should distinguish between internal delays and external capacity constraints, so that improvement work is directed to the right part of the system. Finally, discharge planning should be embedded into hip fracture governance alongside established measures such as time to surgery, orthogeriatric review, mobilisation, and bone-protection treatment.

Limitations

This study has limitations. Its single-centre before-and-after design means that causality cannot be confirmed. The observed improvement may have been influenced by secular trends, seasonal variation, staffing changes, community capacity, or other initiatives taking place at the same time. The sample size was modest, which limited the ability to detect differences in readmission, mortality, and subgroup outcomes. MFFD documentation may also have varied between clinicians, and reasons for discharge delay may not have been coded consistently. In addition, we did not assess patient-reported outcomes, carer experience, post-discharge function, or community-service capacity. Finally, because the intervention was introduced as a bundle, the relative contribution of each individual component cannot be determined.

## Conclusions

At a major trauma centre, introduction of a multidisciplinary discharge-readiness bundle was associated with fewer delayed discharges and a shorter length of stay after neck of femur fracture surgery. The bundle reframed discharge planning as an active part of the care pathway, beginning at admission and progressing alongside medical and surgical recovery, rather than as an administrative step left until the end of the admission. These improvements were achieved without an observed increase in 30-day readmission or inpatient mortality.

Future work should prospectively evaluate this pathway, include patient-centred outcomes, and link acute hospital data with community-service data to better understand the remaining delays related to social-care and rehabilitation capacity.
